# Current Controversies in Diagnosis and Management of Cleft Palate and Velopharyngeal Insufficiency

**DOI:** 10.1155/2015/196240

**Published:** 2015-07-26

**Authors:** Pablo Antonio Ysunza, Gabriela M. Repetto, Maria Carmen Pamplona, Juan F. Calderon, Kenneth Shaheen, Konkgrit Chaiyasate, Matthew Rontal

**Affiliations:** ^1^Speech and Language Pathology Services, Ian Jackson Craniofacial and Cleft Palate Clinic-Neuroscience Program, Beaumont Health System, 3535 West 13 Mile Road, Royal Oak, MI 48073, USA; ^2^Center for Genetics and Genomics, Facultad de Medicina, Clinica Alemana, Universidad del Desarrollo, Avenida Las Condes 12438, 7710162 Santiago, Chile; ^3^Hospital Padre Hurtado, Avenida Esperanza 2150, San Ramon, 8880465 Santiago, Chile; ^4^Cleft Palate Clinic, Hospital Gea Gonzalez, Calzada Tlalpan 4800, 14000 Mexico City, DF, Mexico; ^5^Ian Jackson Craniofacial and Cleft Palate Clinic, Neuroscience Progra, m Beaumont Health System, 3535 West 13 Mile Road, Royal Oak, MI 48073, USA

## Abstract

*Background*. One of the most controversial topics concerning cleft palate is the diagnosis and treatment of velopharyngeal insufficiency (VPI). *Objective*. This paper reviews current genetic aspects of cleft palate, imaging diagnosis of VPI, the planning of operations for restoring velopharyngeal function during speech, and strategies for speech pathology treatment of articulation disorders in patients with cleft palate. *Materials and Methods*. An updated review of the scientific literature concerning genetic aspects of cleft palate was carried out. Current strategies for assessing and treating articulation disorders associated with cleft palate were analyzed. Imaging procedures for assessing velopharyngeal closure during speech were reviewed, including a recent method for performing intraoperative videonasopharyngoscopy. *Results*. Conclusions from the analysis of genetic aspects of syndromic and nonsyndromic cleft palate and their use in its diagnosis and management are presented. Strategies for classifying and treating articulation disorders in patients with cleft palate are presented. Preliminary results of the use of multiplanar videofluoroscopy as an outpatient procedure and intraoperative endoscopy for the planning of operations which aimed to correct VPI are presented. *Conclusion*. This paper presents current aspects of the diagnosis and management of patients with cleft palate and VPI including 3 main aspects: genetics and genomics, speech pathology and imaging diagnosis, and surgical management.

## 1. Imaging Procedures for the Assessment of Velopharyngeal Function during Speech: Multiplanar Videofluoroscopy and Intraoperative Videonasopharyngoscopy

At the present time, the combination of videonasopharyngoscopy (VNP) and multiplanar videofluoroscopy (MPVF) is the procedure of choice for assessing velopharyngeal function during speech. VNP can provide images of the entire vocal tract in motion during speech production also known as articulation. MPVF provides X-ray images of the vocal tract during this same function [1–4].

The earliest recorded examination of the velopharyngeal sphincter in motion during speech was reported by Hilton in 1836. The earliest radiographic assessment of the velopharyngeal valve appeared in 1909 [5]. Since those earlier reports, after extensive research and technological advancement, VNP and MPVF are today the state of the art for the examination of the upper vocal tract during speech including the velopharyngeal sphincter. Although some centers use only the lateral or sagittal view of the soft palate and posterior pharyngeal wall during speech, in combination with VNP [6], several reports have emphasized the importance of a three-dimensional conceptualization of velopharyngeal seal during speech, that is, to study the velopharyngeal valve from the coronal, sagittal, and axial planes [1–3].

One of the most important elements of velopharyngeal closure is lateral pharyngeal wall motion. The use of only a sagittal view precludes the observation of this element. Furthermore, due to velar overlapping it is relatively frequent that lateral pharyngeal wall motion cannot be appropriately examined by VNP.

The advantage of VNP is that the soft tissue structures of the vocal tract, especially the velopharyngeal sphincter, can be examined at different levels and from different angles. However, it is not possible to make real-size measurements of the spaces and structures because the image becomes enlarged as the scope approaches the target and it becomes smaller when the scope is being pulled away. In contrast, MPVF provide visualization of the velopharyngeal sphincter in motion during speech by creating an X-ray image through tissues. Using digital imaging, MPVF can also provide real-size measurements by a ratio of selected distance markings and pixels.

The vocal tract can be conceived as the enclosed space between the vocal cords at the glottis and the lips. The space includes the hypopharynx or laryngopharynx, the oropharynx, the rhinopharynx, the nasal cavities, and the oral cavity. Several structures limit the space at different levels including the epiglottis, the walls of the pharynx at different levels, the base of the tongue, the dorsal aspect of the tongue, the soft palate or velum, the hard palate, the alveolar arches, and the lips. Furthermore, movements of the jaw can significantly modify the shape and dimensions of the vocal tract during speech production [5].

The sound sources of speech production are pulses of air expelled into the vocal tract by adduction and vibration of the vocal folds. These pulses are denominated vocal sources. The pulses resonate on different structures along the vocal tract, also called articulators. The complex and subtle modifications of the fundamental sound wave, also called harmonics, provide the acoustic characteristics to the sounds in order to make them intelligible, that is, to become phonemes or speech sounds. Vocal tract motion for speech production is regulated by the central nervous system [5–7].

One of the acoustic characteristics of speech is nasal resonance. Resonance refers to the quality of the speech sounds by the participation of the nasal cavities into the articulation process. The velopharyngeal sphincter regulates the communication between the nasal cavities and the rest of the vocal tract. Thus, by creating a complete seal, the velopharyngeal sphincter can enhance intraoral pressure for the production of specific phonemes (plosives, fricatives, and affricates). Also, by increasing or decreasing coupling between cavities, the velopharyngeal sphincter balances resonance during speech. An increased nasal resonance is called hypernasality, whereas a decreased nasal resonance is called hyponasality [7, 8].

There have been several attempts to standardize the results of VNP and MPVF. Since VNP does not provide real-size measurements, the use of movement ratios of each one of the structures of the velopharyngeal sphincter seems to be the best approach for describing velopharyngeal function. Although interobserver reliability of the movement ratios has been shown to be statistically nonsignificant, they have become a useful clinical estimate. Movement ratios have also been used for describing velopharyngeal function as observed by MPVF. However, at the present time digital imaging can provide real-size measurements with significant interobserver reliability [1–5, 9].

The velopharyngeal sphincter is comprised by the velum in the anterior aspect, the lateral pharyngeal walls and the posterior pharyngeal wall. Velum motion during speech is accomplished by the synergic movement of the musculus uvulae and the levator veli palatini muscle. Movements of lateral and posterior walls are the result of the action of the superior pharyngeal constrictor. All velopharyngeal muscles are innervated by the IX and X cranial nerves [5].

The adenoid or pharyngeal tonsil is a pad of lymphoid tissue on the posterior wall at the rhinopharynx. The size of the adenoid pad significantly modifies velopharyngeal space.

VNP and MPVF during speech should be video-recorded with sound. In order to perform an adequate assessment of velopharyngeal function it is essential to have a solid Speech and Language Pathology background. The selection of an appropriate speech sample is extremely important. Moreover, before attempting to visualize the velopharyngeal valve it is necessary to rule out the presence of compensatory articulatory errors. When compensatory errors occur, velopharyngeal motion globally decreases providing a “false” picture of the velopharyngeal sphincter in action [5, 7].

The examiner must assure that the patient is capable of repeating at least isolated words including fricative and plosive phonemes with adequate articulation placement [7].

Velopharyngeal inability for creating an efficient seal during speech is defined as velopharyngeal insufficiency (VPI). The etiology of this dysfunction may be anatomical as in cases of cleft palate or functional as in cases of myasthenia gravis among other neuromuscular disorders [5, 12].

VPI is an eminently clinical diagnosis. Although Nasometry can provide objective data for evaluating nasal resonance (measured as mean nasalance), the diagnosis of VPI is made by an accurate Speech and Language Pathology evaluation including assessment of articulation placement and manner, oral, and pharynx examination through direct vision and palpation of the hard and soft palate. The main use of the imaging procedures is to individually study velopharyngeal motion of each of the structures, velopharyngeal closure pattern, and size and shape of the gap during speech. Also, it is extremely important to determine if there is a significant risk of airway obstruction considering the surgical procedure which will be performed in order to correct VPI. If such risk is detected, tonsils and adenoid should be surgically removed in preparation for velopharyngeal surgery. A few months after this initial procedure, after adequate tissue healing, surgical correction of VPI can be performed with the best probability of success [1, 3, 13, 14].

VPI can occur as a consequence of a cleft palate as an isolated malformation. However, cleft palate can be associated with a congenital syndrome and in some of these cases surgical management has to be modified. The most common syndrome associated with cleft palate is 22q11.2 microdeletion syndrome. In these cases, it is common to find internal carotid arteries with an abnormally midline displacement at the level of the pharynx. VNP has been reported as useful for detecting pulsations on the lateral and posterior pharyngeal walls and this information can lead to the diagnosis of a syndromic cleft palate [15–18].

The most important element for performing successful imaging procedures of the velopharyngeal sphincter is to achieve adequate compliance from the patient in order to perform a complete and accurate evaluation. A recent study assessed whether postponing VNP until the patient is in the operating room under light preoperative sedation could result in similar or even better outcomes than performing both procedures during the presurgical evaluation. The goal of this project was to reduce the inefficiencies of the presurgical evaluation and expedite care for patients who require velopharyngeal surgery and may be intolerant of VNP. As mentioned herein, velopharyngeal surgery aims to correct residual velopharyngeal insufficiency in patients with cleft palate. Preoperative MPVF and VNP provide data for customizing the surgical technique for correcting VPI. Although the combination of these procedures during the presurgery appointment is considered the gold standard for the presurgical evaluation of VPI, it has been demonstrated that, without sedation, MPVF is significantly better tolerated than VNP [9, 19].

The study protocol included performing a detailed clinical speech and vocal tract assessment, a Nasometry during the repetition of standardized and phonetically balanced reading passage (“The Rainbow Passage”), and a sustained/e/sound and a sustained/a/sound. Also, a MPVF for assessing the velopharyngeal sphincter function during speech as well as the possible risk of obstruction after velopharyngeal surgery is performed. According to the results of these initial evaluations, velopharyngeal surgery was indicated. If a risk of obstruction was detected, an adenoidectomy and tonsillectomy were scheduled in preparation of the velopharyngeal surgery.

VNP was performed when the patient was already in the operating room under the effect of preoperative light sedation. Patients were still capable of cooperating during the procedure by repeating the speech samples presented by the examiner. The surgical procedure was customized individually in each case, according to the findings of the previous MPVF and the “intraoperative” VNP. That is, the height at which the pharyngeal flap or flaps should be located and the symmetry or skewedness and the width of the flap or flaps were tailor-made depending on the specific structural and anatomical characteristics of each case. It should be pointed out that it has been demonstrated that the anatomical and motion patterns of the velopharyngeal sphincter during speech significantly vary from individual to individual [1, 2, 14].

VPI is present in every case with an ouvert cleft palate and some cases with submucous cleft palate. After palatal repair, 20–40% of the cases persist with residual VPI which requires a second surgical procedure. At present time, pharyngeal flaps and sphincter pharyngoplasties are the two surgical procedures of choice for correcting residual velopharyngeal insufficiency [1, 14, 20].

It has been described that VNP and MPVF can provide anatomical and dynamic data which can be used to select and design the most efficient procedure for correcting velopharyngeal insufficiency. Moreover, several reports support the statement that when surgery is customized individually according to the findings of videonasopharyngoscopy and videofluoroscopy, the speech outcome is significantly improved [1, 3, 9, 14].

Although the combination of VNP and MPVF is considered as the current standard of care protocol for performing preoperative planning in preparation for velopharyngeal surgery, there are important differences between these two procedures. VNP provides* in vivo* view of the velopharyngeal sphincter motion during speech. It does not involve radiation and there are practically no possible complications. However, VNP is poorly tolerated by most young children aged four to ten or by persons with disabilities.

More specifically, due to discomfort during this procedure, without sedation it is sometimes difficult to obtain appropriate compliance from these patients. Thus, the preoperative VNP evaluation must often be rescheduled within this population due to procedural noncompliance and patient discomfort. Furthermore, in several of these cases, the total time necessary to appropriately complete the procedure is frequently prolonged. Before starting the procedure, it usually takes longer time to explain the procedure to younger children and to try to convince them to comply with the instructions. During the procedure, if the patient is not being cooperative, it takes longer to obtain all the necessary data for an adequate selection, planning, and design of the surgical treatment. As a result of these situations, resources can be potentially wasted and quality of care may be potentially reduced.

In contrast, MPVF also provides visualization of the motion of the velopharyngeal valve during speech. This procedure involves a small amount of radiation for the patient. Nonetheless, it has been demonstrated that MPVF is significantly better tolerated than VNP [9, 19].

A prospective, cross-sectional, open clinical trial was carried out in order to study whether a VNP performed in the operating room under light preoperative sedation could provide an adequate assessment of velopharyngeal motion during speech for planning operations aimed to correct VPI. Also, the study would assess comfort of this intraoperative procedure as compared with a VNP performed as an outpatient procedure. The study protocol was reviewed and approved by the Internal Review Board of Beaumont Health, Royal Oak, MI. It was anticipated that patients who received the VNP under light preoperative sedation in the operating room would have similar surgical outcomes concerning correction of VPI and including postoperative mean nasalance scores and clinical nasal emission assessment as compared to patients who received both preoperative evaluation procedures prior to the operation. Additionally, it was anticipated that procedure compliance and patient comfort would be improved in patients who received delayed VNP.

The goal was to reduce the inefficiencies of the preoperative evaluation and expedite care for patients who require velopharyngeal surgery and may be intolerant of VNP. In other words, the study tested whether postponing VNP until the patient would be in the operating room under light preoperative sedation could result in similar or even better outcomes than performing both procedures during the preoperative evaluation.

In 1979, Shprintzen et al. reported that varying the type of insertion of the flap into the palate, postoperative flap width could be tailored to the size of the gap in the velopharyngeal sphincter [21].

Gart and Gosain [1] used pharyngeal flaps and sphincter pharyngoplasties which were performed according to findings of VNP and MPVF. They found that these imaging procedures can provide anatomical and physiological data for planning the surgical procedures for correcting hypernasality. Ysunza et al. [19] reported that MPVF is a safe and reliable procedure for assessing adenoid hypertrophy and velar movement in children.

In 2009, Marsh reported that while the diagnosis of velopharyngeal dysfunction is made by auditory perceptual evaluation, identification of the mechanism of dysfunction requires instrumental visualization of the velopharyngeal port during specific speech tasks. Matching the specific intervention for correcting velopharyngeal dysfunction with the abnormal anatomy can maximize the result while minimizing the morbidity of the intervention [22].

Ysunza et al. found that MPVF seems a reliable method without serious complications for evaluating adenoid hypertrophy and velopharyngeal closure in children, besides being a procedure that is better tolerated, as compared with VNP [19].

The main barrier which may impede widespread implementation of “intraoperative” VNP is that VNP equipment may not be available inside the operating room in all centers.

For the project addressing the use of intraoperative VNP for planning velopharyngeal surgery, twenty patients aged 4–10 years of age or otherwise impaired were studied. All patients presented with residual VPI after surgical repair of a cleft palate. The following questions were presented to the patient and family:

1. How would you/or your child rate the discomfort your child experienced during the MPVF done on (date of the procedure)? (a) No discomfort; (b) mild discomfort; (c) moderate discomfort; (d) extremely uncomfortable.
2. How would you/or your child rate the discomfort your child experienced during the VNP performed in the operating room on (date of the procedure)? (a) No discomfort; (b) mild discomfort; (c) moderate discomfort; (d) extremely uncomfortable.

The results of the survey were analyzed. The VNP which were performed in the operating room were considered as “no discomfort” by 16 (80%) of the patients. It seems reasonable to assume that none of these patients even remembered the procedure. The remaining 4 (20%) patients considered the procedure as “minor discomfort.” It should be pointed out that it was difficult to interpret this finding.

It was questionable if the patients actually vaguely remembered the procedure or they just replied randomly. However, it is evident that none of the patients (whether remembering the procedure or not) considered the procedure as “moderately” or “extremely” uncomfortable. Moreover, if the “intraoperative” VNP is compared with the MPVF which is performed as an outpatient without any sedation, a Mann-Whitney *U* test demonstrated a nonsignificant difference between procedures (VF = 15 (75%), “no discomfort,” and 5 (25%), “minor discomfort” (*P* > 0.9)). Data from the previous report [19] mentioned herein about MPVF were similar: VF = 71%, “no discomfort”; 29%, “minor discomfort.”

In contrast, data from the previous report concerning VNP in outpatients without sedation demonstrated that the procedure is significantly less well tolerated: “no discomfort” = 0; “minor discomfort” = 20%; “moderate discomfort” = 70%; “extremely uncomfortable” = 10%.

The questionnaire was also applied to 20 additional patients who underwent the VNP as outpatients during the last year here in our center. It should be pointed out that these patients were not included in the protocol. They were referred from another center and their surgical procedures were not performed here. The age range was not the same as in the protocol but the difference between median ages was nonsignificant. (a Mann-Whitney *U* test *P* > 0.05). Results were also similar as described herein: “no discomfort” = 0; “minor discomfort” = 17%; “moderate discomfort” = 71%; “extremely uncomfortable” = 12%.

As far as the effectiveness of the “intraoperative” VNP for assessing velopharyngeal closure and how useful the data was for tailoring the pharyngeal flap, the surgical procedure completely corrected hypernasality (as measured by Nasometry-Nasalance) in 18 out of 20 patients (90% success rate).

The height of implantation of a pharyngeal flap on the posterior pharyngeal wall is one of the key elements for a successful outcome. A relatively high pharyngeal flap decreases the risk of postoperative airway obstruction, specifically sleep disordered breathing. Also, the lateral borders of a high pharyngeal flap can more efficiently help to seal the velopharyngeal sphincter by contacting the lateral walls at motion during speech.

The height of the implantation of the flap was measured by postoperative MPVF using the level of the hard palate as reference. In the 20 patients who underwent a pharyngeal flap operation which was tailored according to findings of a preoperative MPVF and an “intraoperative” VNP, the mean distance between the hard palate and the flap was 2.5 mm. This distance was compared with the findings of a postoperative MPVF of 20 patients who had undergone a pharyngeal flap operation which was performed elsewhere without preoperative imaging procedures. The mean distance of the flaps in this group of patients was 10 mm. It should be pointed out that 2 of these patients presented with clinical data suggestive of sleep disordered breathing. 

*Conclusion.* Surgical treatment of residual VPI after initial cleft palate repair should not be performed without individual careful planning. VNP and MPVF provide the necessary information for the appropriate planning of operations aimed to correct residual VPI. MPVF is better tolerated than VNP, especially by young children. Performing MPVF as an outpatient procedure and delaying VNP till the patient is in the operating room under the effect of preoperative sedation seems a safe and reliable sequence for planning operations for restoring efficient velopharyngeal closure during speech.

## 2. Speech and Language Pathology Treatment of Articulation Disorders Associated with Velopharyngeal Insufficiency in Patients with Cleft Palate

Patients with cleft palate (CP) may be at risk for speech disorders. Certain articulation disorders are generally regarded as compensatory behaviors secondary to velopharyngeal insufficiency (VPI). These errors include dysfunction not only of the velopharyngeal sphincter, but also of the entire vocal tract and higher levels of articulation control in the central nervous system [23].

Cleft palate speech is associated with VPI and includes deviations in the resonance such as hypernasality or hyponasality, errors that are obligatory with VPI like nasal emission and weak pressure consonants, and compensatory articulation [11].

Hypernasality is excessive nasal resonance during production of vowels, usually caused by VPI, and is relatively frequent in these patients. Some patients especially with repaired cleft palate can also show other resonance alterations like a reduction in normal nasal resonance resulting from nasal blockage called hyponasality (i.e., turbinate hypertrophy, nasal septum deviation, and obstructing pharyngeal flap). Also, in cleft palate speech mixed resonance can be heard and it is when hypernasality and hyponasality occur simultaneously. Finally, Cul-de-sac resonance is a variation of hyponasality associated with tight anterior nasal constriction producing a muffled quality to sounds [10].

Nasal emission is the escape that accompanies the production of consonants requiring high oral pressure (plosives, fricatives, and affricates). It is considered an obligatory error if it is consequence of VPI or the presence of fistulae and requires physical management.

Compensatory Articulations (CA) are abnormal patterns of articulation and occur when the articulating structures are placed inappropriately resulting in one sound substituting another. CA affects intelligibility and requires speech therapy for correction. This disorder can be considered as a phonologic disorder since it may initially occur as a consequence of the cleft, but the errors become incorporated into the child's developing rule system producing a phonologic disorder [24]. Different authors have described different types of CA in cleft palate speech including glottal stop, pharyngeal stop, pharyngeal fricative, pharyngeal affricate, posterior nasal fricative, middorsum palatal stop, and nasal fricative (see Table 1).

The results from the speech evaluation will help to establish the goals for intervention. However, correcting CA should be the main focus in speech intervention in patients with cleft palate.

Speech intervention with a phonetic approach considers articulation learning as a specific time of motor learning that occurs at a peripheral level. Consequently, intervention procedures are based on the notion that articulation errors are due to faulty control of the articulators [25]. In contrast, in a phonologic approach children must learn more than articulatory patterns associated with words. They must learn a complete phonology—rule system—that occurs at a central level and requires cognitive-phonological processing [26].

When two different approaches for speech intervention in children with cleft palate and CA were compared—phonetic versus phonologic—the total time of speech intervention necessary for correcting CA was critically reduced when a phonological approach was used [27].

Because the phonological system is integrated with the language system it is also suggested that the language of children with CA should also be assessed. Hoffman in 1992 [26] stated that children's speech sound production and perception errors are related not only to phonological knowledge but also to higher organizational levels of language processing. Other researchers have also identified language problems in children with cleft palate, including syntax (i.e., grammar), morphology, and vocabulary [28, 29]. Moreover, Pamplona and Ysunza, 2000, studied the relationship between CA and the child's language system. They found that children with CA showed linguistic performance below the expected level according to chronological age. Hence, if we assume that children presenting with CA show linguistic organization disorders, an intervention aimed to correct CA should include a simultaneous approach for enhancing cognitive linguistic organization.

Whole language principles state that phonologic information should not be separated from the other areas of language, such as pragmatics or syntax. Thus, the same activity provides several pieces of information about all areas of language, including phonology. Phonologic information, such as articulation, is provided in an integrated way within a significant event such as storybook reading or symbolic play with the use of abstract and complex levels of language that leads to a coherent and structured discourse [30].

Storybooks are a perfect context for stimulating cognitive and language development in children [24]. Stories have all the elements of the narrative and discourse structure including the relationships that provide order and structure such as temporality, causality, or perspective. Also, they provide stability for seeing those relationships and for working on specific language needs such as articulation patterns of words and/or sounds. Other activities that promote working with speech and language in an integrated way are art, cooking, music, or symbolic play [31]. 

*Conclusion.* Intervention for cleft palate speech should emphasize working with articulation within a whole event. For articulation, focusing on the target sounds during a naturalistic situation such as storybook reading by analyzing words or patterns could help the child to match events with words and sounds for improving articulation and developing phonemic awareness. This would facilitate generalization of articulation into connected speech.

## 3. Genetics and Genomics of Cleft Palate

### 3.1. Palatogenesis and Cleft Palate

#### 3.1.1. Development of the Primary and Secondary Palate

Craniofacial development in mammals is a highly regulated, complex process that occurs as a result of the interaction of many different gene products with environmental factors during early embryonic development.

The palate is a structure formed by bony or osseous and muscular tissues. The* primary palate* (also named* premaxilla*) is the most anterior aspect of the hard or osseous palate, beyond the anterior incisive foramen. The* secondary palate* is formed by the dental arches, excepting the incisive portion, the palatine osseous vaults, and the muscular soft palate or velum. These structures serve as a structural separation between the oral and the nasal cavity [32].

Development of the human face begins around week four of embryogenesis [33]. It starts with the formation of five facial prominences that surround the mouth: a rostral frontonasal prominence, a lateral pair of maxillary prominences, and a caudal pair of mandibular prominences. These structures are populated by cranial neural crest cells, which are particularly sensitive to perturbations of certain pathways that have been implicated in the occurrence of clefting syndromes [32].

Development of the palate begins around week 6 of gestation in humans (embryonic day E12 in mice) and is identifiable by the appearance of palatal primordia at the lateral edges of the maxillary prominences. These processes fuse coordinately to form the nostrils, upper lip, and palatal shelves that will, in turn, give origin to both primary and secondary palate [32].

Later (week 9 in humans, E14-15 in mice), the bilateral palatal shelves that had been allowed to elevate above the dorsum of the tongue as a consequence of the lengthening of the mandible towards the front of the face grow toward each other and will form the midline edge seam that consists of epithelial cells that produce a glycoprotein coat and desmosomal junctions that allow cell-cell interactions [34].

The mechanism that promotes sealing of the midline seam is unclear. Three hypotheses have been proposed: programmed cell death or apoptosis of cells in this region, migration to other regions of the palatal region (oral or nasal), and finally epithelial-mesenchymal transition (EMT). The process of palatal fusion is completed by week 12 in humans (E16 in mice) and is marked by the complete disappearance of the midline edge seam. Successive ossification that will form the hard (primary) palate will only occur upon successful completion of the fusion process.

Palatal fusion and ossification will ultimately give rise to a normal palate. This process is regulated by a myriad of factors, including growth factors, proteins from the extracellular matrix (ECM), and adhesion molecules. Because of this, palatal fusion has been under intense scrutiny in regard to its implications in the etiology of cleft palate (CP). We next summarize the role of selected genes and pathways implicated in palatal fusion and in the development of CP.

#### 3.1.2. Molecular Pathways Involved in Palatogenesis

*The TGFβ Pathway.* This family of secreted proteins is perhaps the most widely studied in palatal development. TGF*β* is a member of a family of growth factors that includes bone morphogenic proteins (BMPs) and activins. Almost every cell in the human body, including epithelial, endothelial, and mesenchymal cells, produces TGF*β* in any of its isoforms (1, 2, or 3) and has receptors for it [35]. TGF*β* activity can have different consequences depending on context. For example, TGF*β* can induce differentiation of stem cells but cell cycle arrest in epithelial cells. The number of genes under control of the TGF*β* pathway varies from a few in pluripotent stem cells to hundreds in differentiated cells [36].

Classical (or canonical) TGF*β*-dependent signaling regulates gene expression by receptor-mediated activation of SMAD transcription factors, including SMAD2 and SMAD3 (receptor-activated SMADS or R-SMADS), which are phosphorylated by the Ser/Thr kinase domain in the type II receptors. Phosphorylation of SMAD2 and SMAD3 leads to association with SMAD4 (co-SMAD) and the subsequent translocation of this complex into the nucleus, where it regulates expression of many target genes in association with other DNA-binding transcription factors.

Extensive cross-talk exists between this pathway and other signaling pathways. For example, activation of the mitogen-activated kinase (MAPK) pathways by other growth factors can lead to inhibitory phosphorylation of SMAD2 and SMAD3 in the regulatory “linker region” and thus inhibition of signaling propagation [36]. Ligand-activated TGF*β* receptors can also activate the MAPKs ERK, JNK, and p38 (examples of so-called noncanonical TGF*β* signaling).

Increases or decreases in the production of TGF*β* are associated to several diseases, such as cancer, fibrotic disease of kidney or skin [37], and those of the connective tissue and cardiovascular system [38–40].

The importance of TGF*β* signaling in palatogenesis has been robustly demonstrated through animal models that lack TGF-*β*3 production (KO mice), which consistently develop cleft palate among other developmental abnormalities [41, 42].

*Sonic Hedgehog (SHH) Pathway.* Sonic hedgehog is one of three members of the family of the Hedgehog proteins (the others are Indian and Desert hedgehog). This pathway is involved in the regulation of cell proliferation, fate specification, and many other developmental processes.* SHH* signaling occurs through binding of the morphogen with its receptor (patched1,* PTCH1*) and subsequent activation of several transcription factors from the Gli family [43]. There is great consensus that* SHH* plays a key role in angiogenesis and vascularization processes in general, and more recently it has also been involved in osteogenesis [44].

*SHH* is expressed in the epithelium on the oral surface, which corresponds to the region where the* rugae palatini *will form. During palatogenesis,* SHH* is involved in cell signaling in both epithelium and mesenchyme highlighting the role this pathway plays in the process of sealing of the secondary palate during embryonic development [45, 46].

Mutations in the* SHH* gene in humans cause holoprosencephaly, which is characterized by abnormal forebrain and facial development, including cleft palate [34]. This is confirmed by generation of mice that are null for* SHH* and that fully recapitulate the phenotype [47].

*WNT Signaling Pathway.* This family of proteins regularly binds to cell-surface receptors of the Frizzled family activating specific patterns of gene expression. It regulates cell-fate determination and tissue patterning during embryonic development [48]. Several proteins from this family are expressed during palatogenesis and genetic variation within these elements is associated with nonsyndromic cleft palate [49–51]. Moreover, there are several mouse models of impairing mutations in several members of the Wnt family that present cleft palate as one of the phenotypes [34].

### 3.2. Syndromic and Nonsyndromic Causes of Cleft Palate

Orofacial clefts are among the most common major congenital anomalies in humans, with average incidences of 1 in 700 live births, and exhibit marked ethnic and geographic differences ranging from 1 in 500 in northern Europe to 1 in 2,500 in Africa [52]. This observation suggests that the contribution of susceptibility genes and/or the frequency of their variants may vary across populations. Based on clinical manifestations, patterns of recurrence, and biological knowledge, orofacial clefts are broadly classified as cleft lip (CL), cleft lip and palate (CLP), and cleft palate (CP), with the first two considered part of a spectrum, distinct from CP. Several studies have documented that, with few exceptions, familial recurrence tends to be specific: for example, in a 35-year population cohort study in Norway, Sivertsen et al. [53] found relative risks of recurrence of 32 (95% CI 24.6–40.3) for any CL (with or without CP), 56 (37.2–84.4) for CP alone, and only 3 (1.3 to 6.7) for “crossover” between both conditions. This emphasizes that there are different causes for these conditions.

Nonsyndromic forms are defined as those in which there are no other evident anomalies or features and account for approximately 50% of cases of CP and 70% of CL and CLP [54]. In contrast, syndromic forms have additional manifestations, and their causative genes, although not completely known, have been better characterized than the nonsyndromic forms, which are more likely of multifactorial etiology with interaction between genetic susceptibility and environmental causes.

#### 3.2.1. Syndromic Causes of Cleft Palate

The designation of orofacial clefts as syndromic is usually based on the presence of additional physical or cognitive abnormalities and, as stated above, it is estimated that approximately 50% of cases of CP are present in the context of a syndrome [54]. A search in commonly used clinical genetic databases shows close to 300 entries for syndromes with CP (excluding CL) in Orphanet (http://www.orpha.net/consor/cgi-bin/index.php) and almost 500 in POSSUM (http://www.possumcore.com/nuxeo/login.jsp). A few of the most common syndromes that include CP include the following.

*Velocardiofacial syndrome* (OMIM # 192439 and 188400) most commonly includes abnormalities of the palate, heart, and recognizable facial features. Initially delineated in 1978, it is caused by a 1.5–3 Mb microdeletion of chromosome region 22q11. With an overall incidence of one in 4000–6000 live births, it is the most common microdeletion syndrome [55]. Palate involvement has a frequency of 60–80% [56] and is usually manifested as cleft of the soft palate, submucous CP, or bifid uvula. Most of the manifestations are thought to arise as a consequence of haploinsufficiency of the TBX1 transcription factor [57].

Other clefting syndromes for which the genetic basis has been identified include* Loeys-Dietz syndrome* (OMIM # 609191), a condition caused by imbalanced TGF*β* signaling and caused by mutations in either subunit of the TGF*β* receptor,* TGFBR1/2* [38].* Shprintzen-Goldberg syndrome* (OMIM # 182212) is caused by mutations in* SKI*, a suppressor of TGF*β* signaling [58].

CP is also a frequent feature of several craniosynostosis syndromes, such as* Apert* (OMIM # 101200),* Crouzon* (OMIM # 123500), and* Saethre-Chotzen* (OMIM # 101400) syndromes, and also in disorders affecting collagen synthesis, like* Stickler* syndrome (OMIM # 108300), that also includes ocular, auditory, and skeletal manifestations.

#### 3.2.2. Nonsyndromic Cleft Palate

A variety of genetic approaches have been used to identify genes and pathways underlying nonsyndromic CP (nsCP), including mouse models, cytogenetic linkage analysis, candidate gene, and genome-wide association (GWAS) studies. Nevertheless, because, among “typical clefts,” nsCP is less common, fewer molecular and epidemiological studies have been performed for this condition compared with CL and CLP [59, 60].

*Candidate Gene Approaches.* As described above, the TGF*β* pathway has been implicated in palatal closure. Consistent with this knowledge, several studies have found evidence of association between nsCP and variants in genes in this pathway as well as in TFG*α* [61–63], but others have not replicated this finding [64]. Similarly, these same studies also explored and identified variants in MSX1 associated with the presence of CP [63, 64]. Msh homeobox 1 (*MSX1*) encodes a member of the muscle segment homeobox gene family. The encoded protein functions as a transcriptional repressor during embryogenesis and may also have roles in limb-pattern formation, craniofacial development, particularly odontogenesis, and tumor growth inhibition. MSX1 is considered a “crossover” gene, since alterations in it have been found in cases of both CP and CLP [65]. Similarly, variants in* FOXE1*, encoding for a transcription factor of the forkhead family, have been associated both with CL and CP in a large study of individuals of European and of Mesoamerican origin [66].

*Genomic Approaches.* As with many other complex disorders, GWAS have been useful in identifying regions in the genome that could potentially harbor causative variants. A search on the NHGRI Catalogue of published genome-wide studies displays 17 loci in regions encoding for 20 genes and 4 loci in intergenic regions associated with clefts, although most of them (if not all) are focused on cleft lip with and without cleft palate [67–69].

Within these regions, some genes previously found to be causative of syndromic forms of CL/P have been confirmed, with* IRF6* (causing van der Woude Syndrome that usually presents with CL) being the most prominent example, but also some other genes in the TGF*β* pathway have also been replicated, such as BMP6 [70].

While GWAS and linkage studies have shed light into genes that play major roles in palatogenesis and defects in this process, they fail (by nature) to point towards mechanisms underlying the phenotype studied. In this regard, the advent of the “-omics” era has allowed researchers to study this complex phenotype from a global perspective, mainly in the form of gene expression profiling.

Jakobsen et al. performed a global profile of gene expression in palatal tissue from patients with nonsyndromic forms of cleft palate and cleft lip and palate, identifying genes such as* OPN* (encoding for osteopontin) and* CCR4* (encoding for chemokine receptor 4) that were differentially expressed between these groups suggesting a role in palatal development [71].

Functional studies in animal models are performed by introducing genetic perturbations of previously identified genes. One of the most widely used animal models for nonsyndromic CP has been the deletion of TGF*β*3 alleles both in haploinsufficient and complete null animals [41]. This has allowed the performing of global analyses that point to the role of new, previously unsuspected genes that could regulate palate development in conjunction with* TGFβ3* [72, 73].

In summary, the availability of genome-wide approaches has helped us understand better the interaction between key players (those genes with major effect in syndromic or nonsyndromic CL/P) and others that, even though could not be identified in case-control or family-based genetic studies, have been functionally validated as previously described. These complementary approaches will greatly advance our understanding of this complex disease.

### 3.3. Teratogens

Several environmental agents and extrinsic agents have been implicated in the pathogenesis of CP, such as maternal smoking and maternal alcohol consumption, as well as lack of use of folic acid supplementation [74]. As with several environmental agents, not all of those exposed manifest the consequences that suggest the existence of a susceptible population. From the epidemiological perspective, this is being explored by means of gene-environment interaction studies (GxE). Several of these have pointed to known genes involved in nsCP, such as TGF-*α*, in which maternal and fetal variants were found to be associated with CP susceptibility in the presence of tobacco exposure during pregnancy in a meta-analysis [75], and variants in TGF*β* associated with submucous clefts and maternal smoking [76]. In addition, other recent candidate and genome-wide GxE studies have revealed other novel candidates that may modulate the effect of tobacco, such as* TBK1, ZNF 236* [77],* SLC2A9,* and* WDR1* [78]. These studies have also pointed to variants in* MLLT3* and* SMC2* related to CP in the context of prenatal alcohol exposure [77]. The findings are relevant, since they may help identify individuals at particularly high risk of developing preventable forms of CP, as well as point to novel pathways involved in orofacial development and palatal closure.

### 3.4. Therapeutic Implications of Molecular Findings

Although surgery is and will continue to be the mainstay for treatment of CP, it is expected that molecular understanding of the processes involved in palate formation will hopefully lead to novel strategies for prevention and treatment of clefts and to diminishing or avoiding its many complications.

Interesting studies in animal models have been published recently in this regard: as mentioned above, there are mice models of Loeys-Dietz syndrome due to deletion of Tgfbr2. These mice develop cleft palate as a result of abnormal TGF*β* activation via TGF-*β* receptor types I and III- (T*β*RI/T*β*RIII-) mediated TRAF6/TAK1/p38 signaling pathway. Two recently published experimental approaches illustrate the feasibility and consequences of modulating the defects caused by the alteration of this pathway.

Iwata et al. [79] described that a loss of Tgfbr2 in mouse cranial neural crest cells results in an elevated expression of TGF-*β*2 and T*β*RIII and defective cell proliferation in the palatal mesenchyme. They found that Tgfb2, Tgfbr1, or Tak1 haploinsufficiency disrupted T*β*RI/T*β*RIII-mediated signaling and rescued the craniofacial anomalies in Tgfbr2 mutant mice, suggesting that modulation of TGF-*β* signaling may be beneficial for the prevention of congenital craniofacial birth defects.

Subsequent work by this group in the same mouse model showed that Tgfbr2 mutant palatal mesenchymal cells accumulate lipid droplets from reduced lipolysis activity and fail to respond to the cell proliferation stimulator sonic hedgehog, derived from the palatal epithelium. Treatment with p38 mitogen-activated protein kinase (MAPK) inhibitor or telmisartan, a modulator of p38 MAPK activation and lipid metabolism, blocked abnormal TGF*β*-mediated p38 MAPK activation, restoring lipid metabolism and cell proliferation activity both* in vitro* and* in vivo*. These results show the influence of TGF*β* signaling on lipid metabolism and the role of metabolic defects in cell proliferation and palate formation. This discovery has broader implications for the understanding of metabolic defects and potential prevention of congenital birth defects [80].

## Figures and Tables

**Table 1:** Types of compensatory articulations.

CA type	Where?	How?	Main substitutes
Glottal stop	Larynx	Glottal closure	Plosives
Pharyngeal stop	Pharynx	Base of the tongue contacts the posterior wall	Velars
Pharyngeal fricative	Pharynx	A fricative made in the pharynx	Sibilant fricatives
Pharyngeal affricate	Larynx Pharynx	Combines fricative and glottal stops	Oral affricates
Posterior nasal fricative	Pharynx	Constriction between the velum and posterior pharyngeal wall	Sibilant fricatives and affricates
Middorsum palatal stop	Midpalatal area	Tongue contact central area of palate	Plosives /t/, /k/, /d/, /g/
Nasal fricative	Nose	Nonturbulent nasal emission	Fricative

Adapted from Peterson-Falzone et al., 2006 [10], and Golding-Kushner, 2001 [11].
